# SG-APSIC1061: First-response infection prevention and control during COVID-19 outbreaks in residential aged-care facilities

**DOI:** 10.1017/ash.2023.66

**Published:** 2023-03-16

**Authors:** Elizabeth Dawson, Elise Campbell

**Affiliations:** Sydney Local Health District, Sydney, Australia; Sydney Local Health District, Sydney, Australia

## Abstract

**Objectives:** COVID-19 has highlighted the importance of the hierarchy of controls and early implementation of transmission-based precautions during outbreaks in residential aged-care facilities (RACFs). The RACF outreach team is a service provided by the Sydney Local Health District (SLHD) that provides RACFs with expert clinical care and advice, along with outbreak management and infection control and prevention education. **Methods:** The RACF outreach team developed 2 unique IPC management tools designed to assist RACFs during the COVID-19 pandemic: (1) the comprehensive initial review and (2) first-responder assessment tool designed to assist the team in identifying high-risk issues during afterhours shifts. The tool reviews 5 key components in outbreak management: screening, PPE usage, resident care, communication and signage, and infection control and prevention. The outreach team provides an IPC report of the comprehensive initial review, which provides site-specific advice regarding zoning, cohorting, implementation of donning and doffing stations, safe staffing and workflows, ventilation, personal protective equipment (PPE) use, and PPE safety. The recommendations supplied in the SLHD IPC report are provided to facilities and are implemented at the facility level. These reviews are followed up in meetings of the outbreak management team conducted virtually via Zoom videoconferencing. These meetings include an RACF senior manager and a representative from the local PHU, the outreach service, the Australian Commonwealth, the Aged Care Commission, an SLHD executive manager, and an infectious diseases practitioner. **Results:** Since the outbreak of the SARS-CoV-2 ο (omicron) variant began in Sydney, Australia, in November 2021, 58 facilities with >2,500 residents have been reviewed, and 57 of these facilities had a COVID-19 outbreak at some point during the pandemic. **Conclusions:** The RACFs in SLHD continue to report death rates <5% among all SARS-COV-2–positive residents.

